# Nematicity and structural strain: a tight connection in Fe-based superconductors

**DOI:** 10.1107/S1600576725007253

**Published:** 2025-09-18

**Authors:** Alberto Martinelli

**Affiliations:** a CNR Institute SPIN, Corso Perrone 24, Genova, I-16152, Italy; Oak Ridge National Laboratory, USA

**Keywords:** superconductor materials, nematicity, line profile analysis

## Abstract

In the present work, the role of microstrain developing in the temperature range above the structural transition in Fe-based superconductors is analysed in depth. Reviewing the results obtained from different compositions, a similar behaviour emerges in all cases. In particular, using an accurate diffraction line broadening analysis it is demonstrated that the tetragonal-to-orthorhombic structural transition occurring on cooling is anticipated by a symmetry breaking developing on the local scale in the tetragonal plane. The increase in microstrain with decreasing temperature in the stability field of the tetragonal phase qualitatively mirrors the development of anisotropy measured in some physical properties, a behaviour ascribed to nematicity. These results demonstrate the tight and delicate interplay correlating structural features on the local scale with transport and magnetic properties.

## Abstract 2

The properties of the nematic phase are analysed from the crystallographic point of view. A close correlation between the microstructural and physical properties of the nematic phase is found.

## 1. Introduction

Fe-based superconductors constitute one of the most widely studied classes of materials in the past decade, ever since the discovery of superconducting transition temperatures up to 26 K in LaFeAs(O_1−*x*_F*_x_*) (Kamihara *et al.*, 2008 ▸). The nematic phase represents one of their most intriguing features. Consistent with experimental observations, the theory of electron nematic order correctly predicts the sequence of structural and magnetic transition in LaFeAsO (Fang *et al.*, 2008 ▸). In its first formulation, this theoretical model was based on localized spin at the Fe sites interacting with neighbouring spins by antiferromagnetic interactions (Fang *et al.*, 2008 ▸). In this scenario, the tetragonal-to-orthorhombic symmetry reduction in LaFeAsO (envisioned as a transition to an electron nematic phase) is driven by magnetism (Fang *et al.*, 2008 ▸). Although the proposed theory correctly describes the sequence of structural and magnetic transitions, it cannot explain why the same structural transition is observed in some cases where magnetism is absent and the ground state is fully superconductive, as in β-FeSe (Margadonna *et al.*, 2008 ▸; Pomjakushina *et al.*, 2009 ▸; Böhmer *et al.*, 2015 ▸), in (Nd_1−*x*_Sr*_x_*)­FeAsO (Kasperkiewicz *et al.*, 2009 ▸) and in a limited compositional range of the SmFeAs(O_1−*x*_F*_x_*) system (Margadonna *et al.*, 2009 ▸; Martinelli *et al.*, 2012 ▸). Subsequently, charge and/or orbital fluctuations were also proposed to play a role in driving the nematic instability (Fernandes *et al.*, 2014 ▸); charge density waves (CDWs) were then detected (Martinelli *et al.*, 2017 ▸; Lee *et al.*, 2019 ▸; Martinelli *et al.*, 2021 ▸), adding a new ingredient in the crystallo-physics of these systems.

By definition, in the nematic phase the symmetry of some physical properties is lower than the symmetry of the underlying crystal structure (Johnston, 2010 ▸; Kivelson *et al.*, 1998 ▸; see also Appendix *A*[App appa]). In terms of group theory, the point group *L* characterizing the symmetry of the tensor physical property is a sub-group of the point group *G* characterizing the crystal structure, *i.e.L* is a subgroup of *G* (*L*
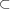
*G*), where every element (symmetry operation) of *L* is an element of *G*. For example, in-plane resistivity anisotropy was observed in a series of detwinned Ba(Fe_1−*x*_Co*_x_*)_2_As_2_ samples well above the structural transition temperature, where a *C*_4_ rotational symmetry in the high-temperature tetragonal phase coexists with the *C*_2_ symmetry of the electronic properties (Chu *et al.*, 2010 ▸).

In fact, what is apparently observed in the nematic phase is opposite to what is postulated by Neumann’s principle, which states that the macroscopic tensor properties of the crystal must have at least the symmetry of the point group of the crystal structure. That is, the point group *G* of the crystal is a sub-group of the symmetry group *L* of any of its physical properties (*G*
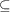
*L* or *L*
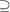
*G*). This implies that, if a crystal is invariant with respect to certain symmetry operations, any of its physical properties must also be invariant with respect to the same symmetry operations. Hence, the in-plane anisotropic behaviour observed in electrical resistivity (a polar tensor property) above the structural transition (in the tetragonal stability field) is clearly opposite to what is predicted by Neumann’s principle.

In this paper, criticalities related to the exact detection and definition of structural and microstructural properties are discussed by reviewing numerous studies conducted on different compositions of phases belonging to the class of Fe-based superconductors. It is suggested that the observed relationships between the symmetry properties of the physical tensors and the underlying crystal structure are consistent with Neumann’s principle when the microstructural properties of these compounds are considered. In this context, we recall that the structure and properties of periodic matter, in whatever dimension and geometry, are strictly related to crystallography (Nespolo, 2015 ▸). For an in-depth treatise on the crystallographic issues examined in the present paper, the reader is also referred to Janovec *et al.* (2013 ▸).

## 2. Method

Details about sample preparation and data collection are found in the articles cited in the text. The diffraction data were acquired during different experimental sessions using the high-resolution neutron powder diffractometer D2B at the Institute Laue Langevin (ILL, Grenoble, France; NPD data) and the X-ray powder diffractometer on the ID22 beamline (old ID31 beamline) of the European Synchrotron Radiation Facility (ESRF, Grenoble, France; XRPD data). The reader is encouraged to consult these cited articles for more precise experimental details.

## 3. Results and discussion

### 3.1. The tetragonal-to-orthorhombic transition in Fe-based superconductors

As a rule, crystal properties are described by a property tensor and the crystal symmetry is defined by a crystallographic point group. The space group type reductions *P*4/*nmm* → *Cmme* and *I*4/*mmm* → *Pmmm* involved in the structural transitions occurring in LnFeAsO (Ln = lanthan­ide), β-FeSe and (*A*,*AE*)Fe_2_As_2_ compounds (*A* = alkaline element and *AE* = alkaline earth element) are characterized by the same point group type dissymmetrization 4/*mmm* ⇓ *mmm* (*G* ⇓ *L*). For this reason, hereinafter only the former transition *P*4/*nmm* → *Cmme* is discussed; the same arguments apply to the latter.

**Figure 1:**
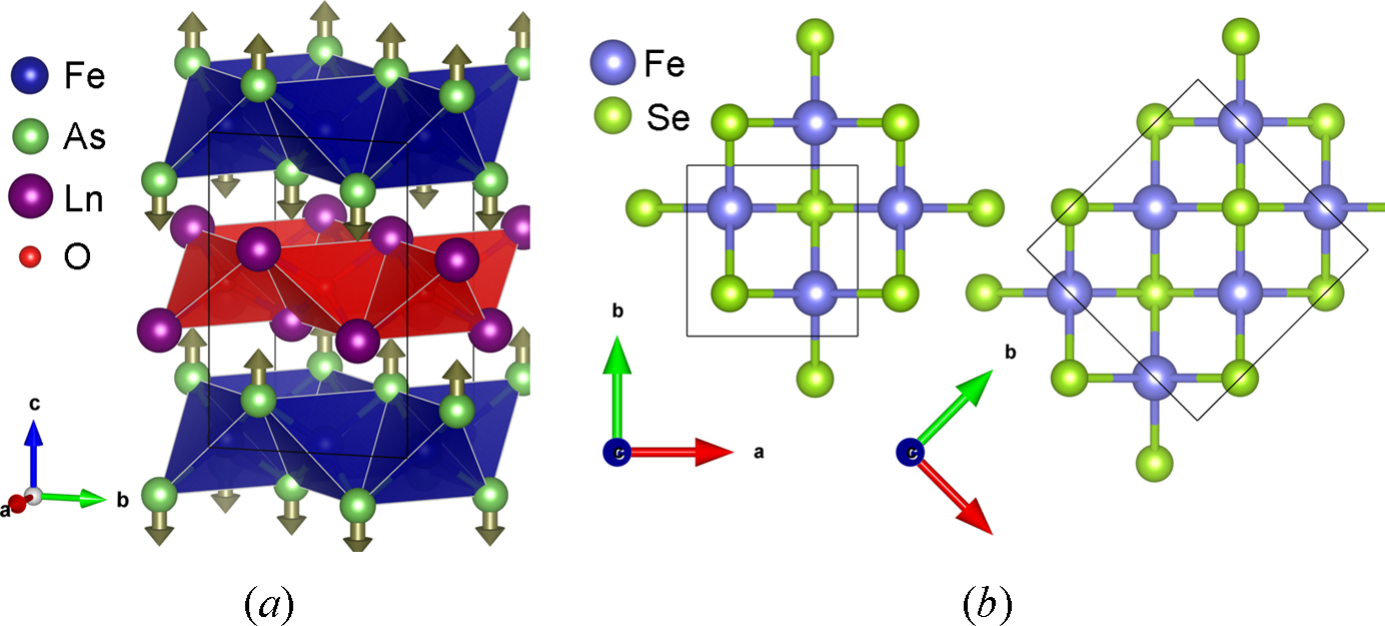
(*a*) Atomic displacement pattern corresponding to the non-symmetry breaking Γ_1_^+^ soft mode in LnFeAsO. (*b*) Relationship between the high-temperature tetragonal and low-temperature orthorhombic unit cells of β-FeSe (selected as representative; grey arrows show the effect of symmetry breaking on the Fe–Fe inter-atomic distances).

The symmetry reduction is classified as ferroic, because the point group type *mmm* is a strict sub-group of 4/*mmm*. In principle, the 4/*mmm* ⇓ *mmm* point group type reduction can be induced by the condensation of the symmetry-breaking Γ_4_^+^ (*B*_2*g*_) soft mode (Salje, 1991 ▸; Martinelli, 2013 ▸). Nevertheless, the displacive Γ_4_^+^ mode is not active at the occupied sites of LnFeAsO, (*A*,*AE*)Fe_2_As_2_ and β-FeSe compounds, and hence the structural transition is not driven by structural degrees of freedom; the non-symmetry breaking Γ_1_^+^ (*A*_1*g*_) is the only soft mode involved in the reduction [Fig. 1 ▸(*a*)]. In fact, the symmetry breaking does not originate from the critical behaviour of phonons associated with the Γ point of the Brillouin zone, but the symmetry reduction is induced by an electronically driven instability (charge, spin or orbital degree of freedom); the structural deformation is therefore the response of the crystal structure to an electronic order parameter that develops on cooling. This is the essential nature of the nematic transition, *i.e.* the dissymetrization does not originate in atomic degrees of freedom. Electronic degrees of freedom (spin, orbital) were proposed to drive the structural transition (Fernandes *et al.*, 2014 ▸); regrettably, up to now the role of CDWs (possibly originating in Fermi surface nesting; Dong *et al.*, 2008 ▸) has been disregarded, although its belated experimental detection (Martinelli *et al.*, 2017 ▸; Lee *et al.*, 2019 ▸; Martinelli *et al.*, 2021 ▸) does demand further study.

In the low-temperature orthorhombic phase, the unit cell is rotated 45° along the *c* axis with respect to the high-temperature tetragonal cell, and the edges of the basal cell are a factor of 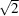 larger [Fig. 1 ▸(*b*)]. The *P*4/*nmm* → *Cmme* transition is equitranslational (*translationengleiche*), as it is described by a representation Γ*_n_* of *G*. As a consequence, twinned crystals form with symmetry reduction, and the primitive unit cells of the *P*4/*nmm* (high-symmetry, HS) and *Cmme* (low-symmetry, LS) phases have the same size, that is the number of atoms per primitive crystal cell remains the same throughout the structural transition. For this reason, no additional optical phonon peaks are observed in the Raman spectra below *T*_s_ (the structural transition temperature). Moreover, theory dictates that the representation Γ*_n_* involved in the dissymetrization determines the principal tensor parameters associated with the primary order parameter; if one of these tensors is a vector (first-rank tensor) or a second-rank tensor, the soft mode is infrared or Raman active in the parent phase, respectively (Tolédano *et al.*, 2006 ▸). In the present case the Γ_1_^+^ representation is associated neither with a vector nor with a second-rank tensor; for this reason, diffraction is the chosen technique to investigate the structural changes in these materials.

Nonetheless, diffraction data must also be analysed with great care. In fact, the observed profile function *h*(*x*) (*i.e.* the diffraction pattern) results from the convolution between the instrumental *g*(*x*′) and the intrinsic diffraction *f*(*x*−*x*′) profile functions:
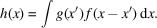
In particular, the *P*4/*nmm* → *Cmme* transition is marked by the selective peak splitting of the {*hhl*} reflections; in the case of a slight structural distortion combined with an inadequate instrumental resolution, the measured profile function can be greatly affected by the contribution of the instrumental profile function, preventing a resolved peak splitting and consequently preventing the correct determination of the crystal structure (and the resulting phase diagrams; Martinelli *et al.*, 2016 ▸).

**Figure 2:**
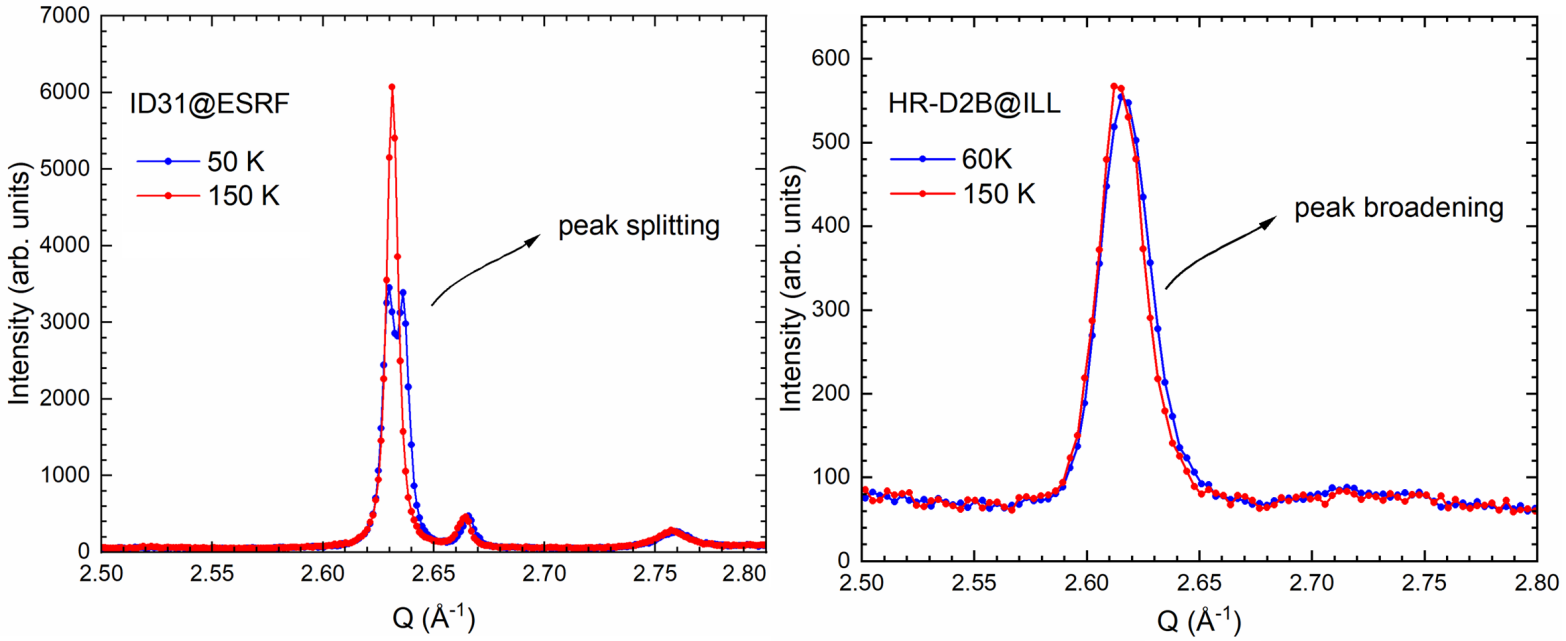
Comparison between (left) the synchrotron X-ray and (right) the neutron powder diffraction patterns collected on the same sample with nominal composition La(Fe_0.90_Ru_0.10_)AsO in the same *Q* range and over the same *T* range.

**Figure 3:**
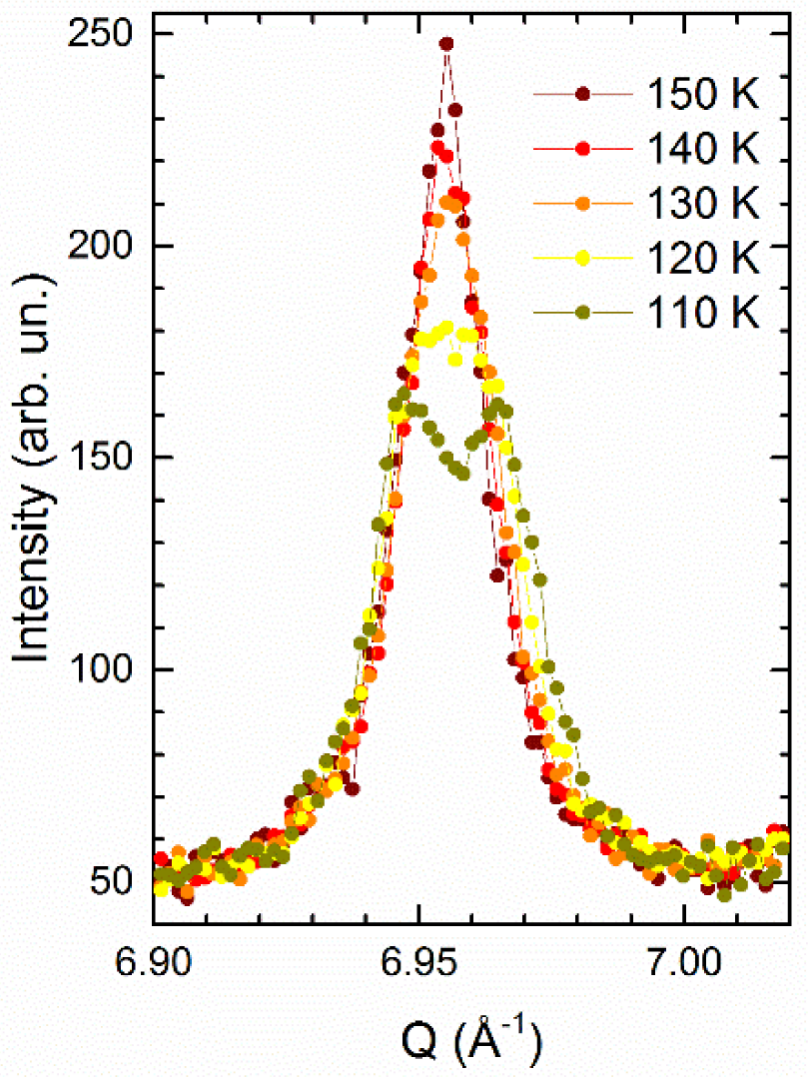
Splitting of the tetragonal 420 diffraction line into the orthorhombic 260 + 620 reflections on cooling in La(Fe_0.90_Ru_0.10_)AsO (NPD data).

This is clearly depicted in Fig. 2 ▸ where the same sample with nominal composition La(Fe_0.90_Ru_0.10_)AsO has been analysed using both a high-resolution synchrotron X-ray powder diffractometer and a high-resolution neutron powder dif­frac­to­meter [for experimental details see Martinelli *et al.* (2013 ▸)]. The diffraction line 112 has been selected because it has a comparable relative intensity in both diffraction techniques. The peak splitting taking place on cooling is evident in the high-resolution XRPD data, whereas the NPD data display only a faint broadening even well below *T*_s_ (∼115 ± 5 K). Hence, in the latter case the structural transition is hidden by the instrumental resolution (actually, some peaks show clear splitting in the NPD data at higher *Q*, marking the structural transition; see Fig. 3 ▸ where the tetragonal 420 diffraction line splits into the orthorhombic 260 + 620 reflections on cooling at 110 K).

**Figure 4:**
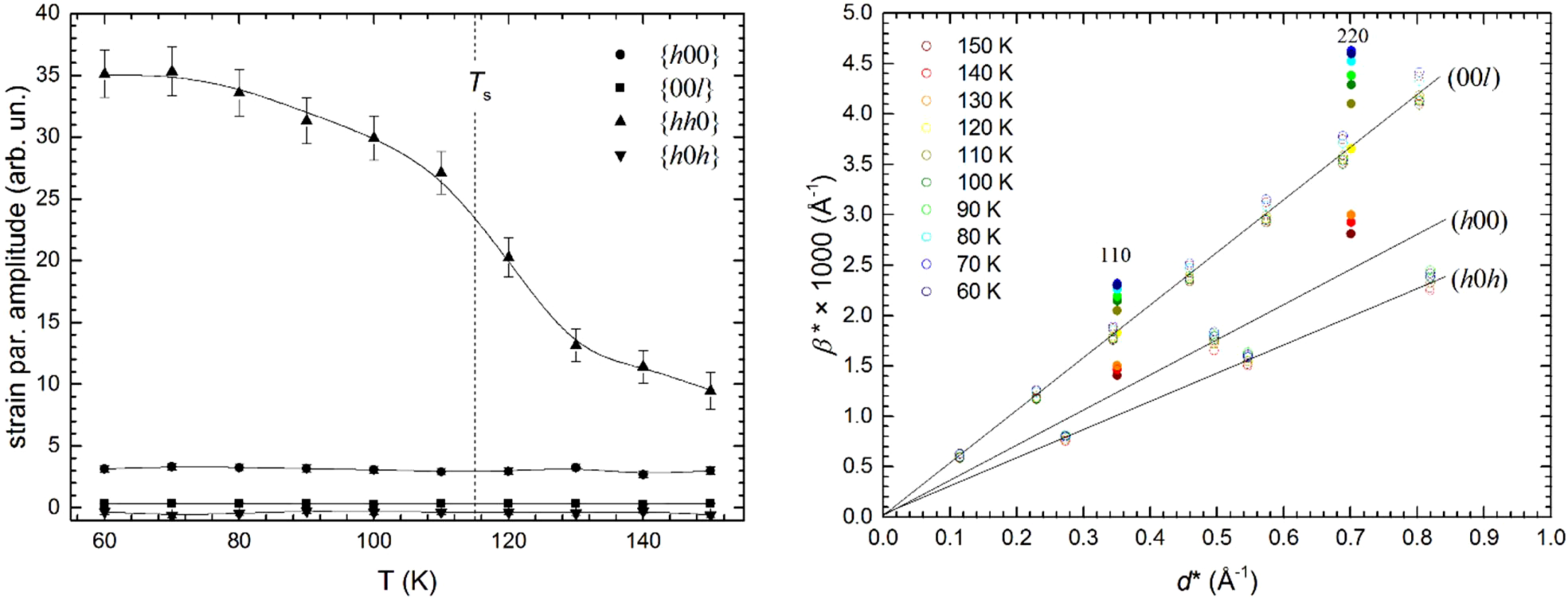
(Left) Evolution with temperature of the anisotropic microstrain representative of the Laue class 4/*mmm* in La(Fe_0.90_Ru_0.10_)AsO (NPD data; lines are guides to the eye). (Right) Superposition of Williamson–Hall plots obtained over the same temperature range. For the sake of clarity, data obtained from (*hh*0) diffraction line data are plotted with full symbols.

Here it is demonstrated that, even when a clear peak splitting cannot be detected, fundamental insights can be gained by an accurate diffraction line broadening analysis (indeed, this technique allowed the first identification of the structural transition in SmFeAsO; Martinelli *et al.*, 2009*a* ▸). In particular, the occurrence of strain broadening in diffraction peaks demonstrates the presence of microstrain, that is, a distribution of interplanar distances around a mean value (Rodríguez-Carvajal *et al.*, 1991 ▸; Stephens, 1999 ▸). Within this scope, Rietveld refinements were carried out using NPD data collected for La(Fe_0.90_Ru_0.10_)AsO; in particular, data exceeding *Q* ≃ 5.75 Å^−1^ were excluded (in order to analyse only the diffraction line broadening and exclude from our analysis the split reflections observed at higher *Q*) and a tetragonal structural model was applied over the whole inspected temperature range. It is apparent that the anisotropic microstrain along {*hh*0} exhibits an abrupt increase some tens of degrees above *T*_s_ and then saturates after the completion of the structural transformation [Fig. 4 ▸(*a*)]. Note that the distortion accompanying the transition occurs exactly along the {*hh*0} crystallographic direction, *i.e.* the diagonal direction of the base square [Fig. 1 ▸(*b*)]. The microstrain increase observed on cooling above *T*_s_ indicates that the tetragonal phase is becoming progressively unstable, although the thermodynamically stable structure is not yet ortho­rhombic. The same scenario stands out by superimposing the Williamson–Hall plots obtained at different temperatures [Fig. 4 ▸(*b*)].

The development of microstrain has a twofold effect. In fact, both (i) the amplitude of the microstrain and (ii) the volume of the strained regions progressively grow when the high-symmetry *P*4/*nmm* phase is cooled down towards *T*_s_. Similar analyses and results on β-FeSe (Martinelli, 2023 ▸) and other LnFeAsO systems (Martinelli *et al.*, 2009*b* ▸; Martinelli *et al.*, 2012 ▸; Martinelli *et al.*, 2013 ▸; Martinelli *et al.*, 2019 ▸; Martinelli, 2019 ▸) have been reported; using a different approach, comparable conclusions were also obtained by analysing BaFe_2_(As_1−*x*_P*_x_*)_2_ single crystals (Kasahara *et al.*, 2012 ▸).

**Figure 5:**
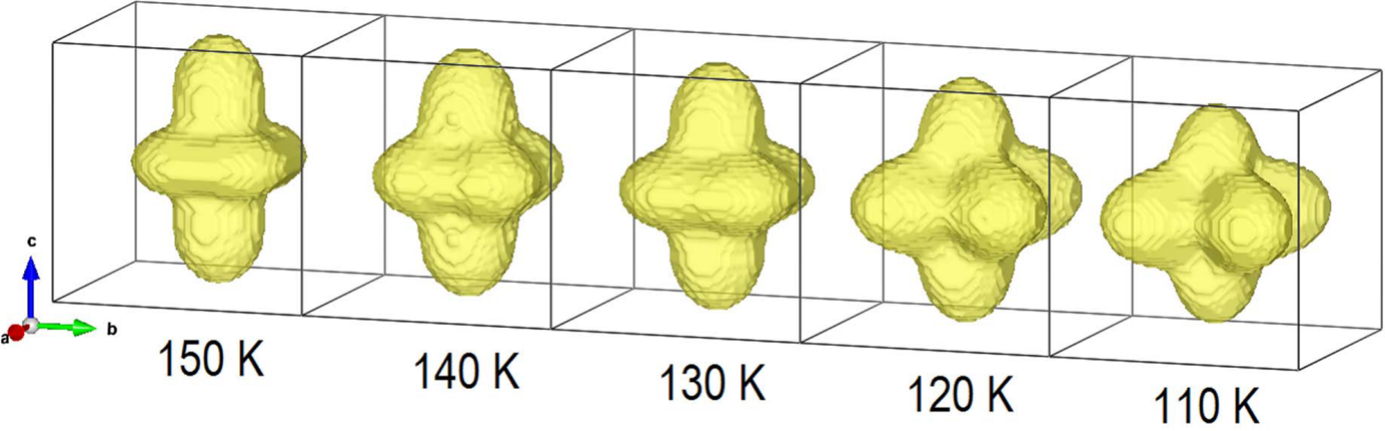
Evolution with temperature of the strain tensor surface characterizing La(Fe_0.90_Ru_0.10_)AsO (obtained using the refined tetragonal anisotropic strain parameters).

Another remarkable result is depicted in Fig. 5 ▸, where the tensor surface representing the structural microstrain exhibits the peculiar morphology theoretically calculated for the 4/*mmm* ⇓ *mmm* point group type dissymetrization (Leineweber, 2011 ▸). In particular, a four-fold tensor surface is theoretically predicted to develop in the *ab* plane for this symmetry reduction, fully consistent with our data. All these results demonstrate that an accurate microstructural analysis can reveal (or at least suggest) the occurrence of a subtle structural transition, even when the instrumental resolution prevents a sharp peak splitting (Fig. 2 ▸).

### 3.2. Microstructural features below the transition temperature

Below the transition temperature, the symmetry reduction is accompanied by the development of a domain structure in the ferroic *Cmme* polymorph (transformation twins) to accommodate the crystal structure change (Ma *et al.*, 2009 ▸; Tanatar *et al.*, 2009 ▸). In particular, the *Cmme* space group type is a maximal non-isomorphic subgroup of *P*4/*nmm* and the transition thus produces a twin domain structure. The dimension and shape of the domains depend on many factors, among which the most relevant are the kinetics of the phase transition and the occurrence of local stresses and defects. The change in the point group symmetry at the phase transition determines the type of domain structure. All domains are characterized by the same crystal structure as the low-temperature polymorph, differently oriented in the different domains. Remarkably, these different domains even exhibit different tensor properties. A ferroelastic domain structure can grow when the structural transition is characterized by a decrease in the independent strain components; in this case, the mechanical strain is different in the different domain states, but by applying a mechanical stress the different orientation states can be switched.

**Figure 6:**
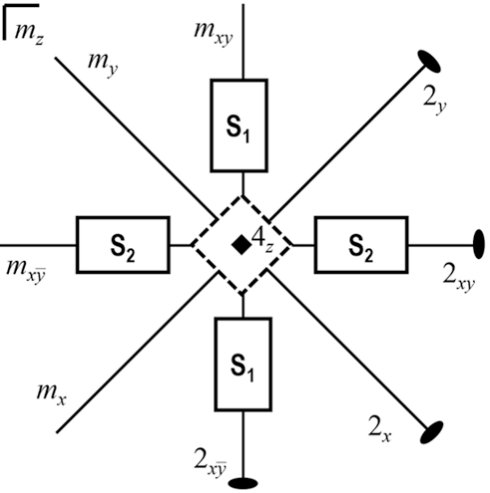
Exploded view of single-domain states **S**_1_ and **S**_2_ (solid rectangles) formed during the transition 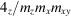 ⇓ 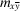. The tetragonal phase is represented by the dashed square (rotated by 45°; see Fig. 1) and possible variants of the orthorhombic phase by rectangles. Symmetry elements of the point group 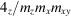 are also drawn.

In the inspected case, the dissymetrization 4/*mmm* ⇓ *mmm* (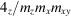 ⇓ 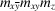) is a ferroic transition accompanied by a spontaneous strain (the part of the strain due entirely to the structural transition; Salje, 1991 ▸), giving rise to ferroelastic domains with different strains. The theoretical analysis of this symmetry reduction (Janovec & Přívratská, 2013 ▸) predicts the occurrence of two principal domain states, **S**_1_ and **S**_2_, related by a symmetry operation suppressed during the transitions (Figs. 6 ▸ and 7 ▸). These domain states correspond to two ferroelastic domain states, in perfect agreement with the domain structures observed in (*A*,*AE*)Fe_2_As_2_ compounds (Ma *et al.*, 2009 ▸; Tanatar *et al.*, 2009 ▸).

As the number of ferroelastic domain states is equal to the number of principal domain states, the 4/*mmm* → *mmm* transition is fully ferroelastic, with the principal domain states **S**_1_ and **S**_2_ characterized by spontaneous strain tensors having different orientation states. Mirror planes of the tetragonal structure which are not present in the orthorhombic structure are permissible domain walls in the twin domain structure (Sapriel, 1975 ▸); in the present case permissible domain walls are thus mirror planes *m_x_* and *m_y_* (Figs. 6 ▸ and 7 ▸).

**Figure 7:**
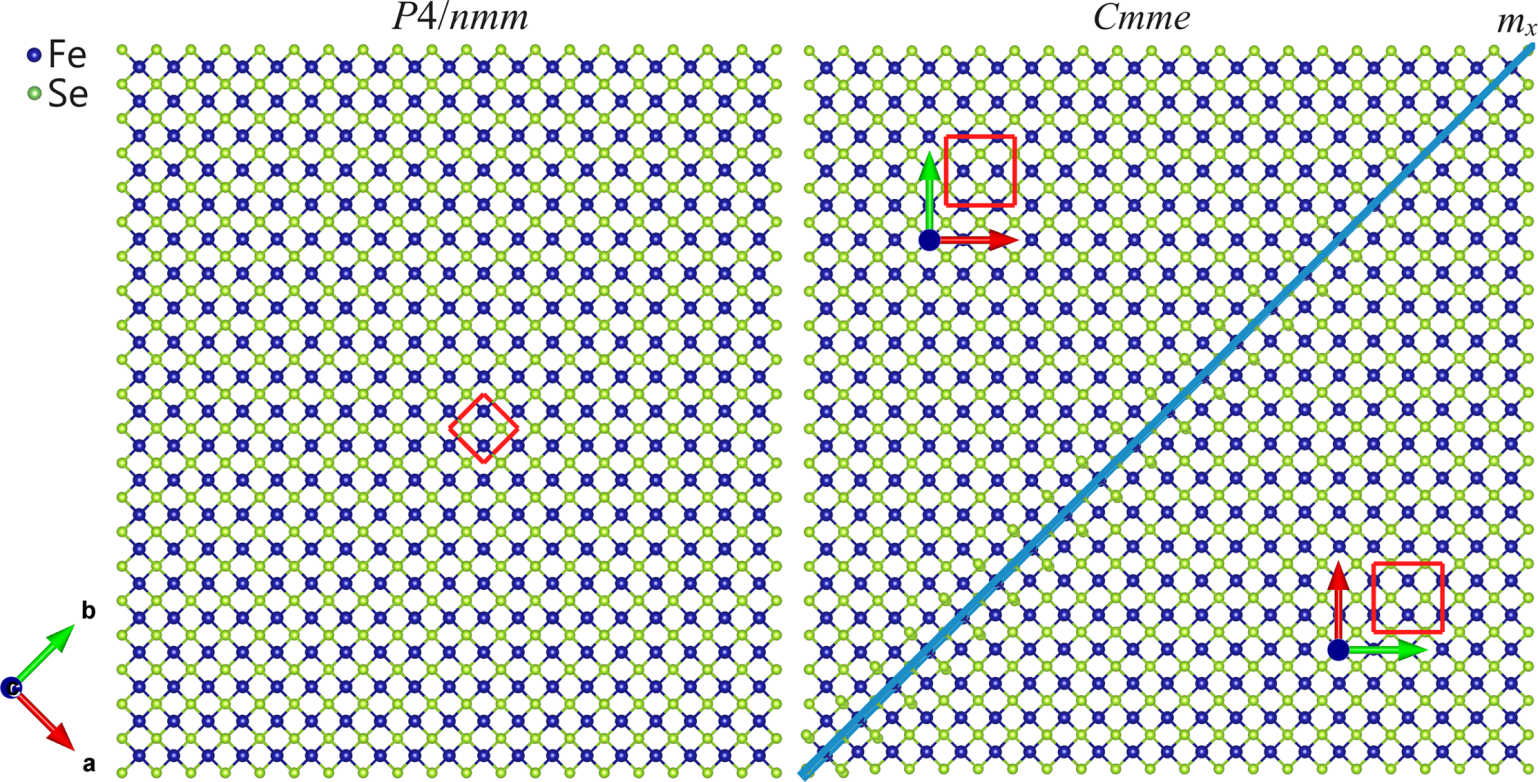
Schematic representations of the atomic arrangement within the [FeSe] layer, (left) in the high-symmetry *P*4/*nmm* phase and (right) in the domain structure of the low-symmetry *Cmme* phase. The point group 4/*mmm* → *mmm* transition gives rise to different ferroelastic domain states (having different orientation states separated by domain walls; in the present case, the mirror plane perpendicular to the tetragonal *x* axis) with different spontaneous strains. Unit cells representative of the tetragonal and orthorhombic structures are highlighted [see also Fig. 1(*b*)].

The strain tensor is a polar symmetric property tensor of second rank; as a general rule, if one second-rank polar symmetric property tensor changes, all other polar symmetric property tensors with rank ≥2 also change (Aizu, 1969 ▸; Aizu, 1970 ▸). In particular, the strain tensor, the electrical conductivity tensor and the magnetic susceptibility tensor are all second-rank tensors and therefore have the same transformationa1 properties; thus their changes are always correlated. As a consequence, when the strain tensor changes the electrical conductivity must also change symmetrically. The domain pair sketched in Fig. 6 ▸ and in the panel on the right-hand side of Fig. 7 ▸ consists of domain states with different orientations, each characterized by its own strain tensor. These domain states can appear with the same probability when related by a symmetry operation suppressed by dis­symetrization, that is a symmetry operation of *P*4/*nmm* but not of *Cmme*. By applying an external mechanical stress, the different states of the domain structure can be oriented along the same direction (detwinning) and hence display the same tensor properties; in this case, distinct resistivities along *x* and *y* can be measured, or are otherwise ideally averaged at about the same value when the two domain states develop with the same probability. The anisotropy measured for the physical property is primarily determined by the symmetry of the [FeSe] layer, rather than by the amplitude of the orthorhombic structural distortion (in the present case, the percentage difference between the cell parameters *a* and *b* in the orthorhombic phase of β-FeSe is less than 0.5%).

### 3.3. Microstructural features above the transition temperature

**Figure 8:**
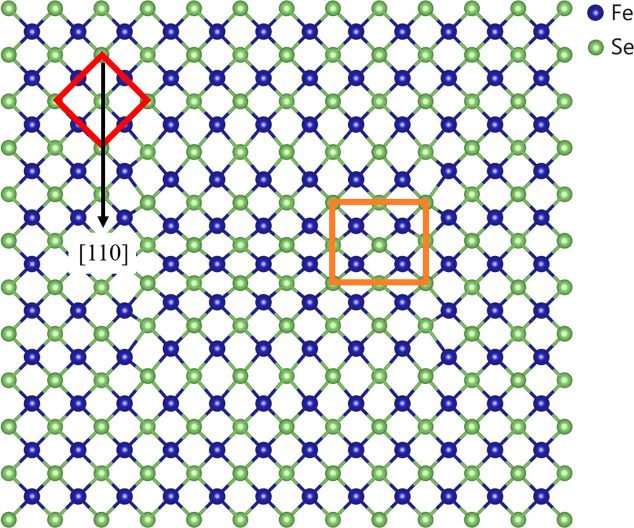
Representation of microstrain within the [FeSe] layer, breaking the 4/*mmm* symmetry on the local scale. In the average tetragonal structure (red cell), locally correlated orthorhombic distortions (orange cell) take place along {*hh*0} [see also Fig. 1(*b*)].

Hitherto the microstructural properties of the low-symmetry *Cmme* phase below *T*_s_ (*T* < *T*_s_) have been examined. It is instructive at this point to analyse the temperature dependence of the microstructural properties (Snyder *et al.*, 2000 ▸) of the HS *P*4/*nmm* phase just above *T*_s_, where the nematic phase is reported to occur. In particular, we have debated the spontaneous strain that measures the amplitude of the deformation of the LS structure with respect to the HS one (the spontaneous strain being null in the HS phase; Salje, 1991 ▸). From now on we analyse the features of the microstrain in the *P*4/*nmm* phase (*T* > *T*_s_), describing the response of the crystal structure of the HS phase to a temperature change. This microstrain originates from the local distortion of the tetragonal structure, *i.e.* the changes in the distances between its various atoms along different crystallographic directions, and occurs on account of static fluctuations and correlations between metric parameters (Rodríguez-Carvajal *et al.*, 1991 ▸). Therefore, the atomic arrangement in the locally distorted regions is not the same as that in an undistorted tetragonal structure, due to the different forces acting on the atoms. The 4/*mmm* symmetry is thus broken on the local scale within some regions of the tetragonal phase, as sketched in Fig. 8 ▸, even though the crystal structure remains *P*4/*nmm* on average. Nonetheless, the interactions between the electrons and the atoms are modified in the distorted regions, thus affecting the electron–structure coupling and transport properties (Millis, 1998 ▸). Fluctuations of the order parameter occur on the local scale on cooling (reaching a critical level as *T*_s_ is approached) and progressively affect the strain tensor. In the present case, the strain is not produced by stress, but it is caused by a temperature change, which is a scalar quantity and hence has no orientation. Consequently, the strain must be invariant with respect to the symmetry of the crystal (Nye, 1957 ▸). For the 4/*mmm* point group the corresponding symmetry-adapted form of the strain tensor is
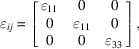
with two independent coefficients. This expression holds when microstrain is negligible in the tetragonal structure.

The temperature evolution of microstrain in the *P*4/*nmm* phase is illustrated in Fig. 4 ▸ and shows a progressive increase in microstrain along {*hh*0} starting some tens of degrees above *T*_s_, *i.e.* in the same temperature range as where nematic properties are typically measured. As mentioned above, the orthorhombic structural distortion accompanying the transition occurs along this same {*hh*0} direction. Consequently, a microstrained tetragonal structure with locally correlated orthorhombic distortions takes place in this temperature range. On cooling/heating, the crystal deformation is described by the thermal expansion 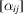, a second-rank tensor relating the strain to the change in temperature:
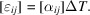
Then, on the local scale and for the orthorhombic distorted regions, the thermal expansion tensor is related to the strain tensor:
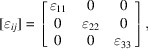
with three independent coefficients conforming to the local symmetry. As all second-rank tensors have the same transformation properties, their changes are thus correlated and always occur simultaneously. Since the strain (a polar symmetric property tensor of second rank) changes with temperature, all other polar symmetric property tensors with rank = 2 (such as electrical conductivity) must transform in the same way. In particular, the development of microstrain along {*hh*0} in the *P*4/*nmm* phase breaks the 4/*mmm* symmetry on the local scale; therefore, the symmetry group characterizing the electrical conductivity accordingly also changes on the local scale and resistivity fluctuations occur along the same tetragonal {*hh*0} crystallographic directions where microstrain develops. This is the reason why resistivity displays different values when measured along different in-plane directions above *T*_s_ in the tetragonal phase of untwinned crystals (Chu *et al.*, 2010 ▸).

Note that the magnitude of ρ_*b*_/ρ_*a*_ for in-plane resistivity of detwinned Ba(Fe_1−*x*_Co*_x_*)_2_As_2_ samples decreases with heating for *T* > *T*_s_; this is exactly the same dependence on temperature as observed for the microstrain along {*hh*0} (Fig. 4 ▸). This feature clearly shows the close relationship and interplay between the microstrain (and the associated strain tensor on the local scale) and electrical resistivity (and more generally between the strain and other physical property tensors with rank ≥2). Similarly, the linear coupling between the microstrain and the nematic order parameter obtained by magnetic torque measurements (Kasahara *et al.*, 2012 ▸) must be ascribed to the fact that the magnetic susceptibility tensor and the magnetic permeability tensor are again second-rank tensors.

A final remark concerns the degree(s) of freedom driving the structural microstrain and dissymetrization. The development of CDWs is consistent with the Fermi surface nesting characterizing these compounds, even though initial investigations ruled out its occurrence on the basis of the erroneous conclusion that a negligible structural distortion takes place on cooling (Dong *et al.*, 2008 ▸). The recent experimental detection of incommensurately modulated phases in these materials (Martinelli *et al.*, 2017 ▸; Lee *et al.*, 2019 ▸; Martinelli *et al.*, 2021 ▸) strongly demands further theoretical investigations into the role played by CDWs in the physics of these materials. It is likely that the observed structural distortion results from an electrostrictive coupling between the strain tensor and the electric field associated with the CDWs. As a rule, the development of the incommensurate state is accompanied by a microstrain-like diffraction line broadening (Leineweber & Petricek, 2007 ▸), a behaviour fully consistent with our microstructural analysis.

## 4. Conclusion

A careful analysis of structural and microstructural properties as a function of temperature reveals a significant evolution of the microstrain in the tetragonal polymorph of Fe-based superconductors. In particular, local distortions are shown by the progressive increase on cooling of the microstrain along {*hh*0} in the tetragonal phase as the tetragonal-to-ortho­rhombic structural transition is approached. Consequently, a microstrained tetragonal structure with locally correlated orthorhombic distortions occurs in this temperature range. As thermal expansion and the associated strain are second-rank polar symmetric property tensors and must conform to crystal symmetry (even on the local scale), their dependence on temperature is expected to symmetrically affect all other polar symmetric property tensors with rank ≥2, such as electrical conductivity and magnetic susceptibility. As a result, the anisotropy of the physical properties observed above *T*_s_ and ascribed to nematicity is actually provided by a diffuse short-range structural distortion breaking the average tetragonal symmetry on the local scale.

Ultimately, this scenario reconciles the apparent violation of Neumann’s principle (stating that *G*
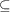
*L*) with the current definition of the nematic phase provided for Fe-based superconductors, *i.e.* a phase with a tetragonal structure but with physical properties characterized by a lower symmetry (*L* 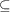 *G*).

## Data Availability

Data supporting the reported results can be accessed upon request.

## Appendix A On the original definition of the nematic phase

In the original formulation of the nematic state applied to a two-dimensional square lattice, it is stated that ‘*the nematic phase breaks the four-fold rotation symmetry of the lattice, but leaves both translation and reflection symmetries unbroken*’ (Kivelson *et al.*, 1998 ▸). Although seldom confused with the concept of structure, the lattice is constituted by an infinite set of integral linear combinations of linearly independent vectors in the vector space. The points of the point lattice thus have a periodic order, they are all equal and they all have the same surroundings. The symmetry group *G* of a square lattice consists of eight mapping elements *g* (identity, one two-fold rotation axis, two four-fold rotation axes and four mirror planes) (Müller, 2013 ▸). Group theory dictates that, to each product *g_i_* × *g_j_* of two elements of a group *G*, there corresponds a unique element *g_k_* of *G*. This means that it is not possible to eliminate the four-fold rotation elements while keeping unchanged the reflection elements. In fact, the product of two reflection planes that are not mutually perpendicular to each other corresponds to a four-fold rotation axis. For example, by multiplying the reflection plane perpendicular to the *x* axis with a diagonal reflection plane, the four-fold rotation element is again generated:

Nonetheless, for a crystal the metric of the unit cell associated with the lattice can be accidentally higher than expected (for example, an orthorhombic crystal structure with a unit cell of lattice parameters *a* = *b*) within certain temperature and/or pressure ranges. In this case, the lattice is promoted to a higher symmetry, but without increasing the underlying symmetry of the structure (Nespolo, 2015 ▸).
